# Prevalence and Influencing Factors of Anxiety and Depression in Patients With Breast Cancer: A Meta-Analysis

**DOI:** 10.62641/aep.v54i2.2167

**Published:** 2026-04-15

**Authors:** Hua Qing, Lina Ye

**Affiliations:** ^1^Emergency Department of West China Hospital, Sichuan University, 610066 Chengdu, Sichuan, China; ^2^Colorectal Cancer Center, Department of General Surgery, West China Hospital, Sichuan University, 610041 Chengdu, Sichuan, China

**Keywords:** breast cancer, anxiety, depression, prevalence, risk factors, mental health

## Abstract

**Background:**

Breast cancer (BC) patients shoulder considerable psychological strain as they traverse the illness trajectory. While anxiety and depression among this population have received substantial research attention, comprehensive global estimates that distinguish anxiety from depression remarkably scarce. Even more understudied is the systematic review of factors unique to each condition. Our meta-analysis was designed to redress these gaps by establishing worldwide pooled prevalence figures for both anxiety and depression among BC patients, alongside mapping their associated risk and protective influences.

**Objective:**

This study aimed to obtain consolidated global estimates of anxiety and depression prevalence within BC populations, and to explore various risk and protective factors that shape these.

**Methods:**

This systematic review and meta-analysis included cross-sectional and cohort studies from multiple databases reporting anxiety/depression prevalence in BC patients. Two investigators independently conducted study selection, data extraction, and quality assessment using the Newcastle-Ottawa Scale (NOS). A random-effects model pooled prevalence estimates; heterogeneity was explored via subgroup analysis and meta-regression. Publication bias was assessed with Egger’s test and funnel plots.

**Results:**

This meta-analysis included 32 studies comprising 21,507 BC patients. The pooled prevalence of anxiety was 35% (95% confidence intervals (CI): 30%–39%), and that of depression was 26% (95% CI: 23%–30%), with significant heterogeneity for both (*p *< 0.001). For anxiety, a high Life Orientation Test-Revised (LOT-R) score was protective, whereas low income was a risk factor. For depression, protective factors included older age, higher income, early tumor stage, and a high LOT-R score. Risk factors were low education, rural residence, disrupted marital status, comorbidities, lack of social support, and a history of mental illness. Sensitivity analysis confirmed that the results of this study were robust; although there was bias in anxiety and depression, its effect is limited after correction.

**Conclusion:**

Anxiety and depression are highly prevalent in breast cancer patients, influenced by distinct sociodemographic and clinical factors, necessitating targeted psychological assessment and intervention.

## Introduction

Breast cancer (BC) is one of the most common malignant tumors among women around the world. In 2022, there were about 2.3 million new cases of BC in the world, and its incidence is still on the rise [1, 2, 3]. In recent years, the prognosis of BC has been significantly improved. In many countries, the 5-year survival rate of patients can reach about 90%, and the 10-year survival rate is about 80% [4]. However, prolonged survival has not significantly improved the mental health of patients. Such patients still face great psychological pressure throughout the process of diagnosis, treatment and rehabilitation. Depression and anxiety are common complications in BC patients [5]. A systematic review shows that the prevalence of psychological distress in adult female BC patients can reach up to 52% [6]. Anxiety and depression not only seriously damage the patient’s quality of life and lead to dysfunction in emotional, social and cognitive domains but are also closely associated with physical symptoms such as pain and insomnia. In addition, such psychological problems can also have an adverse impact on treatment compliance, disease prognosis and even long-term survival rate [7, 8]. Therefore, it is crucial to estimate the prevalence of anxiety and depression in this population and identify their key influencing factors.

Previous research has extensively investigated risk factors for BC-related psychological distress, anxiety, and depression, including sociodemographic characteristics, disease- and treatment-related features, psychological factors, and social environment factors. A meta-analysis among Chinese BC patients indicated that individuals aged <40 years, unmarried, with low education levels, low income, advanced tumor stage, those undergoing modified radical mastectomy, with comorbidities, and receiving chemotherapy had a significantly elevated risk of depression [9]. Another meta-analysis in BC patients demonstrated that factors including education level, tumour stage, income level, insurance, and employment status were closely associated with psychological distress [6]. Although the number of systematic reviews and meta-analyses on psychological distress, anxiety, and depression in BC patients has increased in recent years, existing studies still have certain limitations: many systematic reviews and meta-analyses treat psychological distress as a composite outcome, failing to distinguish anxiety from depression as distinct dimensions of emotional disorders [6]; some studies are confined to specific countries or regions, lacking comprehensive global data integration [10, 11]; other studies focus primarily on whether anxiety and depression contribute to cancer recurrence or suicide, without separately analyzing the prevalence and associated factors of these two emotional problems [5, 12]. Therefore, this study conducted a meta-analysis of the prevalence of anxiety and depression and their associated factors influencing factors in BC patients worldwide. This study aimed to clarify the pooled prevalence level of anxiety and depression and related influencing factors, in order to help clinicians accurately identify the characteristics of high-risk groups and promote the implementation of early screening and targeted psychological interventions.

## Materials and Methods

### Literature Search Strategy

This meta-analysis was carried out in accordance with the guidelines of systematic review and PRISMA. The research was retrieved electronically in the following databases: PubMed, Embase, Web of Science and Cochrane Library. The retrieval time limit covered each database from the construction of the library to December 2025. The search term was set to: (“Breast Neoplasms” OR “Breast Cancer”) AND (“Anxiety” OR “Depression” OR “Psychological Distress”) AND (“Prevalence” OR “Incidence” OR “Epidemiology”).

This study took the PubMed database as an example, and the detailed retrieval strategy was as follows: (“Breast Neoplasms”[MeSH Terms] OR “breast neoplasm”[Title/Abstract] OR “breast cancer”[Title/Abstract] OR “mammary cancer”[Title/Abstract] OR “breast carcinoma”[Title/Abstract] OR “breast tumor”[Title/Abstract] OR “breast tumor”[Title/Abstract]) AND (“Anxiety”[MeSH Terms] OR “Depression”[MeSH Terms] OR “Anxiety”[Title/Abstract] OR “depressi”[Title/Abstract] OR “psychological distress”[Title/Abstract] OR “emotional distress”[Title/Abstract] OR “mood disorder”[Title/Abstract] OR “mental health”[Title/Abstract]) AND (“Prevalence”[MeSH Terms] OR “Prevalence”[Title/Abstract] OR “Incidence”[Title/Abstract] OR “Rate”[Title/Abstract] OR “Epidemiology”[Title/Abstract] OR “Frequency”[Title/Abstract] OR “Occurrence”[Title/Abstract] OR “Burden”[Title/Abstract]) AND (“Risk Factors”[MeSH Terms] OR “risk factor”[Title/Abstract] OR “influencing factor”[Title/Abstract] OR “determinant”[Title/Abstract] OR “predictor”[Title/Abstract] OR “associated factor*”[Title/Abstract]).

### Inclusion and Exclusion Criteria

The inclusion criteria were as follows: (1) study design: cross-sectional studies, or a cohort study that provides baseline data on the prevalence of anxiety and/or depression in BC patients; (2) study population: pathologically confirmed BC patients, age ≥18 years, regardless of nationality, race and treatment stage; (3) outcome indicators: using validated scales (such as Hospital Anxiety and Depression Scale (HADS), Generalized Anxiety Disorder 7-item scale (GAD-7), Patient Health Questionnaire-9 (PHQ-9), Self-Rating Anxiety Scale (SAS), Self-Rating Depression Scale (SDS)) to clearly report the prevalence of anxiety or depression, provided sufficient data for prevalence calculation and/or associated factor analyses.

The exclusion criteria included: (1) review, case report and conference abstract literature; (2) research on optical extraction or calculation of prevalence data; (3) research groups that included other cancer patients or non-cancer groups, and the relevant data of BC patients could not be extracted alone; (4) studies with duplicate publication or overlapping data, such studies retained the largest sample size or the most comprehensive information; (5) studies with incomplete data; (6) studies rated as low quality (i.e., with a NOS score of <4 stars [13, 14]).

### Literature Screening and Data Extraction

This study used EndNote X9 software (Clarivate, Philadelphia, PA, USA) for literature management. The two researchers completed the literature screening independently, and the differences arising in the screening process were resolved through discussion and consultation. Literature screening was divided into two stages: in the first stage, duplicate literature was eliminated, and the title and abstract of the literature were reviewed to complete the preliminary screening; in the second stage, the full text was obtained that potentially met the standard literature, and after detailed evaluation, the final literature was determined. The researchers used a pre-designed data extraction table to independently extract the following information: the first author, the year of publication, the country of the study, the type of research design, sample size, mean age of the patient, the tumor stage, the stage of treatment, the anxiety/depression assessment tool, the number of positive cases or prevalence of anxiety/depression, and statistically significant associated factors.

### Quality Assessment

The methodological quality of cohort studies was assessed using the Newcastle-Ottawa Scale (NOS) [13], and the quality of cross-sectional studies was evaluated using the NOS adaptation for cross-sectional studies (NOS-XS) [14]. Both scales employ a nine-star scoring system. According to the total number of stars awarded, studies were classified as having low (0–3 stars), moderate (4–6 stars), or high (7–9 stars) methodological quality, corresponding to high, moderate, and low risk of bias (RoB). Two researchers independently evaluated study quality, and any discrepancies were resolved through discussion and consensus.

### Statistical Analysis

Stata 18.0 software (StataCorp LLC, College Station, TX, USA) was used for statistical analysis. A single-arm meta-analysis of proportions was performed to calculate the pooled prevalence rate along with its 95% confidence intervals (CI) based on the number of events and the total sample size reported in each study. Given the anticipated clinical and methodological heterogeneity, the DerSimonian-Laird random-effects model was applied. Heterogeneity was assessed using the I^2^ statistic (with I^2^ >50% indicating significant heterogeneity) and Cochran’s Q test. To explore potential sources of heterogeneity and examine associated factors, the following analyses were conducted based on the extracted data: (1) Subgroup analysis: prevalence rates were compared across groups defined by pre-specified characteristics (e.g., geographical region, screening instrument); (2) Meta-regression for single-group rates: covariates were performed for factors reported as statistically significant in two or more studies to assess their contribution to the pooled prevalence. When the direction of the direction of effect for a given factor was inconsistent across studies, effect size were transformed to ensure consistent directionality. The robustness of the findings was examined using sensitivity analysis by iteratively removing individual studies. Publication bias was assessed using funnel plot visual inspection and Egger’s linear regression test (*p* value < 0.05 indicates the existence of publication bias).

## Results

### Literature Screening Process and Characteristics of Included Studies

**Fig. 1.**
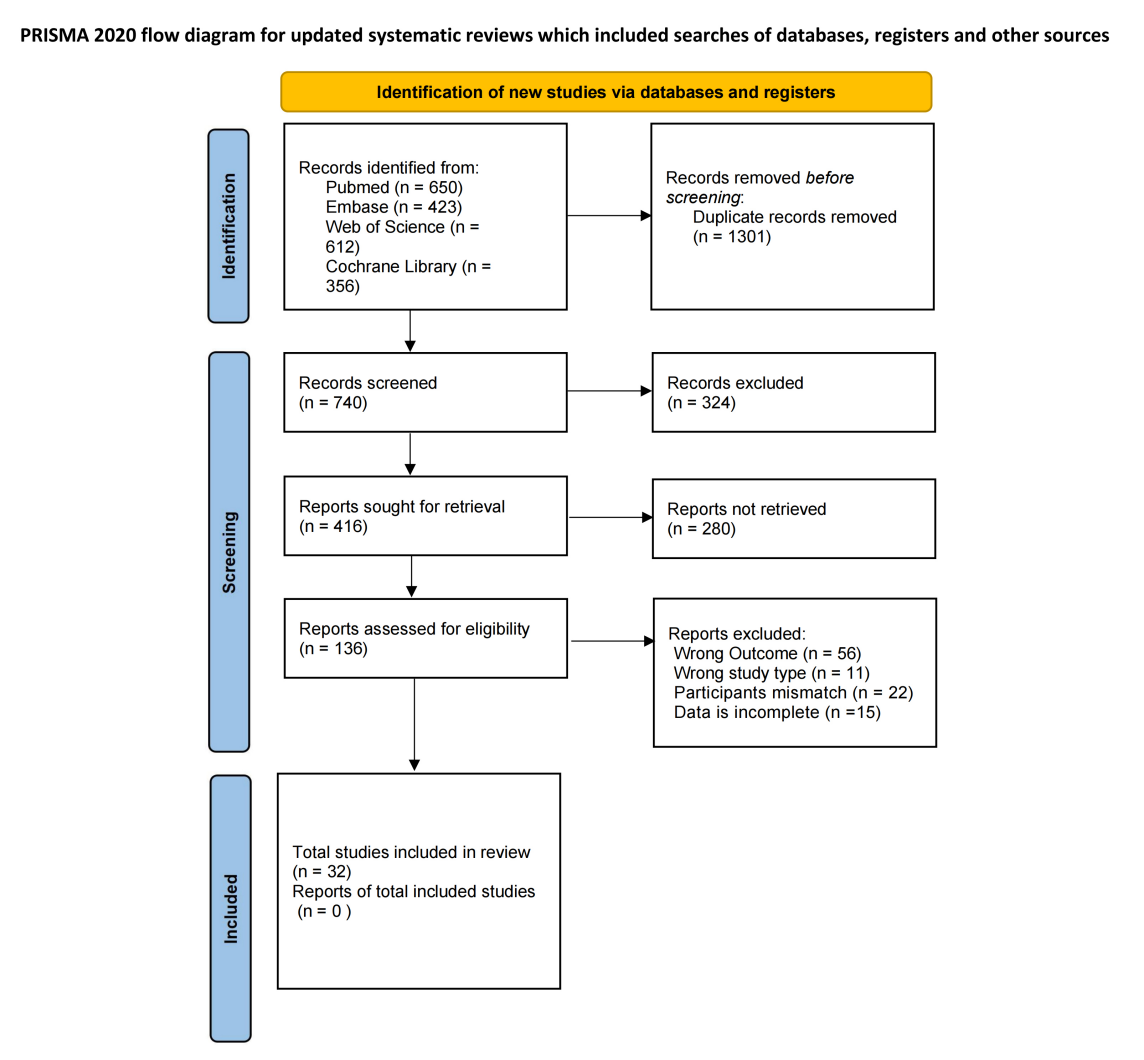
**PRISMA flow chart**. PRISMA, Preferred Reporting Items for Systematic Reviews and Meta-Analyses.

A total of 2041 potentially relevant literatures were obtained through the preliminary search. After eliminating 1301 duplicate documents through EndNote X9 software, the remaining 740 documents entered the title and abstract screening stage. At this stage, a total of 324 literature that did not meet the inclusion criteria were excluded. We then attempted to retrieve the full texts of the remaining 416 potentially eligible records. Of these, 280 reports were excluded due to lack of availability or unavailable full texts. Consequently, 136 reports were successfully obtained and subjected to a detailed eligibility assessment. Finally, 32 studies were included, involving a total of 21,507 BC patients. For the detailed literature screening process, please refer to Preferred Reporting Items for Systematic Reviews and Meta-Analyses (PRISMA) flow diagram in Fig. 1.

The included studies were published between 2001 and 2025 and originated from multiple countries, including Australia [15], Spain [16], Nigeria [17, 18], Japan [19, 20], China [21, 22, 23, 24, 25, 26, 27, 28, 29], Ghana [30], Norway [31, 32], the United Kingdom [33], Greece [34, 35], Pakistan [36], Denmark [37], Tanzania [38], Germany [39], the United States [40, 41], India [42], Iran [43], Malaysia [44], Puerto Rico [45], and South Korea [46].

The most frequently utilized instrument for assessing anxiety was the HADS [15, 16, 24, 25, 30, 31, 32, 35, 39, 40, 44], followed by the GAD-7scale [21, 26, 36, 41, 42]. Similarly, the HADS was also the most common tool for evaluating depression [15, 16, 24, 27, 30, 31, 32, 35, 39, 40, 44], followed by (PHQ-9, PHQ-2) [21, 26, 34, 36, 41, 42] and the Beck Depression Inventory (BDI) [23, 37, 46].

Quality assessment using the Newcastle-Ottawa Scale (NOS) indicated that 19 studies were of high quality (score ≥7 stars) [15, 16, 18, 20, 22, 23, 24, 26, 29, 31, 32, 33, 35, 37, 39, 41, 43, 44, 45], and 13 studies were of moderate quality (score 5–6 stars) [17, 19, 21, 25, 27, 28, 30, 34, 36, 38, 40, 42, 46]. All studies meeting the other eligibility criteria had a NOS score of 5 stars or higher (i.e., all were of moderate or high quality). Therefore, no study was excluded solely based on the quality assessment threshold of ≤4 stars for low quality. The detailed characteristics of the included studies are presented in Table 1 (Ref. [15, 16, 17, 18, 19, 20, 21, 22, 23, 24, 25, 26, 27, 28, 29, 30, 31, 32, 33, 34, 35, 36, 37, 38, 39, 40, 41, 42, 43, 44, 45, 46]).

**Table 1.** **Basic Information of Included Studies**.

	Study	Country	Number of participants	Prevalence (Anxiety/Depression) (%)	Type of research	Tool	Cases (Anxiety/Depression) (n)	NOS
1	Osborne 2003 [15]	Australia	731	45/12	Cross-sectional studies	HADS	328/88	7
2	Puigpinós-Riera 2018 [16]	Spain	2235	48.6/15	Cohort study	HADS	1086/335	7
3	Fatiregun 2016 [17]	Nigeria	200	19/-	Cross-sectional studies	SCAN	38/-	5
4	Akechi 2001 [19]	Japan	148	-/5	Cross-sectional studies	HADS, MAC	-/7	6
5	Chen 2021 [21]	China	834	15.5/21.6	Cross-sectional studies	PHQ-9, GAD-7	129/180	6
6	Kugbey 2022 [30]	Ghana	205	48.5/37.3	Cross-sectional studies	HADS, PDRQ-9	99/76	5
7	Mao 2025 [22]	China	360	-/38.61	Cross-sectional studies	PHQ-9	-/139	7
8	Schou 2004 [31]	Norway	165	26.0/9.0	Cohort study	HADS	43/15	9
9	Faye-Schjøll 2019 [32]	Norway	293	26.3/9.6	Cohort study	HADS	77/28	9
10	Okamura 2005 [20]	Japan	50	-/22.0	Cross-sectional studies	SCID, MAC	-/11	8
11	Hopwood 2010 [33]	UK	2208	32.4/12.0	Cohort study	HADS	705/260	7
12	Konstantinos Tsaras 2018 [34]	Greece	152	32.2/38.2	Cross-sectional studies	PHQ-2, GAD-2	49/58	5
13	Popoola 2012 [18]	Nigeria	124	-/40.3	Cross-sectional study	MINI	-/50	7
14	Qiu 2012 [23]	China	505	-/20.59	Cross-sectional study	BDI, MINI, HAMD	-/95	7
15	Sharif 2025 [36]	Pakistan	96	41.7/79.2	Cross-sectional study	GAD-7, PHQ-9	40/76	6
16	Christensen 2009 [37]	Denmark	3343	-/13.7	Cohort study	BDI-II	-/458	8
17	Msenga 2025 [38]	Tanzania	384	44.8/50.5	Cross-sectional studies	HADS	172/194	6
18	Chen 2009 [29]	China	1400	-/26	Cohort study	CES-D	-/364	8
19	Mehnert 2008 [39]	Germany	1083	38.2/22.2	Cross-sectional studies	HADS	414/240	7
20	Lan 2022 [24]	China	290	35.2/44.1	Cohort study	HADS	102/128	7
21	Trevino 2020 [40]	USA	1085	30.8/11.7	Cross-sectional studies	HADS	334/127	6
22	Husain 2024 [42]	India	192	46.4/29.7	Cross-sectional study	GAD-7, PHQ-9	89/57	5
23	Su 2017 [25]	Taiwan, China	300	-/8.33	Cross-sectional study	MINI	- /25	6
24	Hessami A 2025 [43]	Iran	283	56.9/46.6	Cross-sectional study	DASS-21	161/132	7
25	Hassan 2015 [44]	Malaysia	205	31.7/22.0	Cross-sectional study	HADS	65/45	7
26	Ayala-Rodríguez 2025 [45]	Puerto Rico	208	14.9/19.2	Cohort study	PHQ-8, GAD-7	31/40	7
27	Reece 2013 [41]	USA	32	15.6/37.5	Cohort study	PHQ-9, GAD-7	5/12	7
28	Wang 2025 [26]	China	504	26.0/37.3	Cohort study	PHQ-9, GAD-7	131/188	7
29	Kim 2008 [46]	South Korea	1933	-/24.9	Cross-sectional study	BFI, BDI, EORTC QLQ-C30, EORTC QLQ-BR23	-/372	5
30	Ge 2024 [27]	China	1613	31.0/21.0	Cross-sectional study	HADS	500/341	6
31	Guo 2023 [28]	China	176	60.2/52.3	Cross-sectional study	DASS-21, Brief COPE	106/92	5
32	Aggeli 2021 [35]	Greece	170	29.4/18.2	Cross-sectional study	HADS	50/31	7

HADS, Hospital Anxiety and Depression Scale; GAD-7, Generalized Anxiety Disorder-7 item scale; PHQ-9, Patient Health Questionnaire-9; PHQ-2, Patient Health Questionnaire-2; BDI, Beck Depression Inventory; MINI, Mini-International Neuropsychiatric Interview; HAMD, Hamilton Depression Rating Scale; SCAN, Schedules for Clinical Assessment in Neuropsychiatry; MAC, Mental Adjustment to Cancer scale; PDRQ-9, Patient-Doctor Relationship Questionnaire-9 items; CES-D, Center for Epidemiologic Studies Depression Scale; ICD-10, International Classification of Diseases, 10th Revision; Brief COPE, Brief Coping Orientation to Problems Experienced Inventory; BFI, Big Five Inventory; EORTC QLQ-C30, European Organization for Research and Treatment of Cancer Quality of Life Questionnaire Core 30; EORTC QLQ-BR23, European Organization for Research and Treatment of Cancer Quality of Life Questionnaire Breast Cancer Module 23; GP, General Practice; GYP, Gynecological Practices; DASS-21, Depression Anxiety Stress Scale-21.

#### Pooled Prevalence of Anxiety and Depression

#### Prevalence of Anxiety

**Fig. 2.**
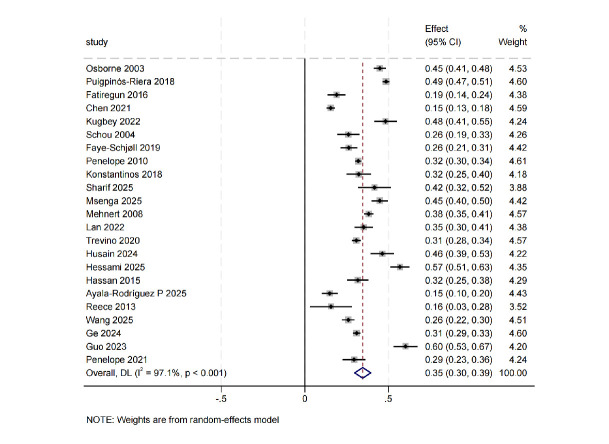
**Prevalence of anxiety**. CI, Confidence interval.

A total of 23 studies reported data on anxiety [15, 16, 17, 21, 24, 26, 27, 28, 30, 31, 32, 33, 34, 35, 36, 38, 39, 40, 41, 42, 43, 44, 45]. The pooled analysis using a random-effects model showed that the prevalence of anxiety symptoms among BC patients was 35% (95% CI: 30%–39%). Considerable heterogeneity was observed across the included studies (I^2^ = 97.1%, *p* < 0.001) (Fig. 2).

#### Prevalence of Depression

**Fig. 3.**
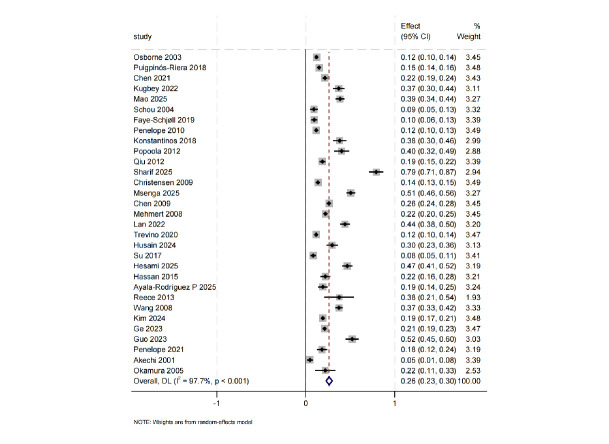
**Prevalence of depression**.

A total of 31 studies reported data on depression [15, 16, 18, 19, 20, 21, 22, 23, 24, 25, 26, 27, 28, 29, 30, 31, 32, 33, 34, 35, 36, 37, 38, 39, 40, 41, 42, 43, 44, 45, 46]. The pooled analysis indicated that the prevalence of depressive symptoms among BC patients was 26% (95% CI: 23%–30%). The heterogeneity among studies was substantial (I^2^ = 97.7%, *p* < 0.001) (Fig. 3).

#### Subgroup Analysis

**Fig. 4.**
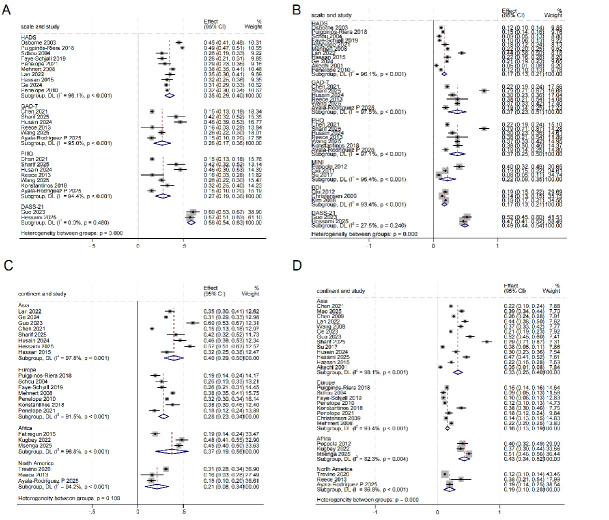
**Subgroup analysis**. (A) Anxiety prevalence as measured by different scales. (B) Depression prevalence as measured by different scales. (C) Anxiety prevalence in different continents. (D) Depression in different continents.

To further explore the potential sources of heterogeneity in anxiety and depression, subgroup analyses were conducted based on assessment tools and geographical regions (Fig. 4). The results were as follows:

Ten studies using the HADS [15, 16, 24, 27, 31, 32, 33, 35, 39, 44] were included (Fig. 4A). The prevalence of combined anxiety was 35% (95% confidence interval: 29%~40%), with substantial heterogeneity (I^2^ = 96.1%, *p* < 0.001). The GAD-7 subgroup included 6 studies [21, 26, 36, 41, 42, 45], reported a pooled prevalence of 26% (95% CI: 17%–36%), with considerable heterogeneity (I^2^ = 95.0%, *p* < 0.001). The subgroup using the Patient Health Questionnaire (PHQ) was included in a total of 7 studies [21, 26, 35, 36, 41, 42, 45]. The prevalence of combined anxiety was 27% (95% confidence interval: 19%~36%), and there was a very high heterogeneity in the group (I^2^ = 94.4%, *p* < 0.001). The DASS-21 subgroup included two studies [28, 43] and showed no significant heterogeneity (I^2^ = 0.0%, *p *= 0.480). The test subgroup differences in anxiety prevalence across assessment tools (*p* < 0.001).

The subgroup using the Hospital Anxiety and Depression Scale (HADS) included a total of 11 studies [15, 16, 19, 24, 27, 31, 32, 33, 35, 39, 44] (Fig. 4B). The pooled prevalence of depression was 17% (95% confidence interval: 13%~21%), with very high within-group heterogeneity (I^2^ = 96.1%, *p* < 0.001). The GAD-7 subgroup comprised 6 studies [21, 26, 36, 41, 42, 45], with a pooled depression prevalence of 37% (95% CI: 23%–51%) and extremely high within-group heterogeneity (I^2^ = 97.5%, *p* < 0.001). The subgroup using the PHQ included 7 studies [21, 26, 34, 36, 41, 42, 45]. The pooled prevalence of depression was 37% (95% confidence interval: 25%~50%), with very high within-group heterogeneity (I^2^ = 97.1%, *p* < 0.001). The subgroup assessed with the Mini‑International Neuropsychiatric Interview (MINI) included 3 studies [18, 23, 25], The pooled prevalence of depression was 22% (95% confidence interval: 9%~35%), with very high within-group (I^2^ = 96.4%, *p* < 0.001). The subgroup using the BDI was included in 3 studies [23, 37, 46], The pooled prevalence of depression was 17% (95% confidence interval: 13%~21%), with very high heterogeneity (I^2^ = 93.4%, *p* < 0.001). The DASS-21 subgroup included two studies [28, 43], with a pooled depression prevalence of 49% (95% CI: 44%–54%) and low within-subgroup heterogeneity (I^2^ = 27.5%, *p* = 0.240). The results of the inter-group heterogeneity test showed that there were statistically significant differences in the prevalence of depression corresponding to different assessment tools (*p* < 0.001).

The Asian subgroup was included 8 studies [21, 24, 27, 28, 36, 42, 43, 44] (Fig. 4C), and the pooled prevalence of anxiety was 40% (95% confidence interval: 29%–50%), with very high within-group heterogeneity (I^2^ = 97.8%, *p* < 0.001). The European subgroup included 7 studies [16, 31, 32, 33, 34, 35, 39], with a combined anxiety prevalence of 28% (95% confidence interval: 23%–34%), and very high heterogeneity in the group (I^2^ = 91.5%, *p* < 0.001). The African subgroup included 3 studies [17, 30, 38], with a pooled prevalence of anxiety of 37% (95% confidence interval: 19%–56%), and very high within‑group heterogeneity (I^2^ = 96.8%, *p* < 0.001). The North American subgroup included 3 studies [40, 41, 45], with a pooled prevalence of anxiety of 21% (95% confidence interval: 8%–34%), and very high within‑group heterogeneity (I^2^ = 94.2%, *p* < 0.001). The results of the inter-group heterogeneity test showed that there was no statistically significant difference in the Prevalence of anxiety corresponding to different geographical regions.

The Asian subgroup included 13 studies [19, 21, 22, 24, 25, 26, 27, 28, 29, 36, 42, 43, 44] (Fig. 4D), The pooled prevalence of depression was 33% (95% confidence interval: 25%~40%), with very high with-group heterogeneity (I^2^ = 98.1%, *p* ≤ 0.001). The European subgroup included 8 studies [16, 31, 32, 33, 34, 35, 37, 39]. The pooled prevalence of depression was 16% (95% confidence interval: 13%~19%), with very high within-group (I^2^ = 93.4%, *p* < 0.001). The African subgroup included 3 studies [18, 30, 38]. The pooled prevalence of co-depression was 43% (95% confidence interval: 34%~52%), with high within‑group heterogeneity (I^2^ = 82.3%, *p *= 0.004). The North American subgroup included 3 studies [40, 41, 45], with a pooled prevalence of depression of 19% (95% confidence interval: 10%~28%), and high within-group heterogeneity (I^2^ = 86.8%, *p* < 0.001). The results of the inter-group heterogeneity test showed that there were statistically significant differences in the prevalence of depression corresponding to different geographical regions (*p* < 0.001).

### Meta-Analysis of Influencing Factors

#### Factors Influencing Anxiety

Age factor: Seven studies analyzed the influence of age on anxiety [15, 16, 32, 33, 36, 40, 45]. Heterogeneity testing showed high heterogeneity between studies (I^2^ = 78.7%, *p* < 0.001). Meta-analysis demonstrated that age was a protective factor for anxiety (OR = 0.90, 95% confidence interval: 0.82~0.98, *p *= 0.011).

Culture level factors: Three studies evaluated the impact of education level on anxiety [15, 33, 40], with high between studies heterogeneity (I^2^ = 89.4%, *p* < 0.001). Low education may increase the risk of anxiety, but the difference was not statistically significant (OR = 1.66, 95% confidence interval: 0.58~4.78, *p *= 0.347).

Tumor staging factors: Four studies investigated the effect of tumor staging on anxiety [15, 17, 36, 38], with high heterogeneity between studies (I^2^ = 82.5%, *p* < 0.001). The effect of early tumor staging on anxiety was not statistically significant (OR = 0.84, 95% confidence interval: 0.42~1.68, *p *= 0.621).

Income level factors: Three studies examined the impact of income level [26, 44, 45], with low heterogeneity between studies (I^2^ = 28.4%, *p *= 0.247). Low income was identified as a risk factor for anxiety (OR = 1.97, 95% confidence interval: 1.39~2.78, *p* < 0.001).

Residence factors: Two studies explored the influence of residence on anxiety [26, 34], with moderate heterogeneity between studies (I^2^ = 70.4%, *p* = 0.066). Living in a rural area may be associated with an increased risk of anxiety, but the difference was not statistically significant (OR = 2.07, 95% confidence interval: 0.84~5.08, *p *= 0.112).

Life Orientation Test-Revised (LOT-R) Score: Two studies assessed the impact of the revised life orientation test score on anxiety [31, 32], and there was no obvious heterogeneity between the studies (I^2^ = 0%, *p *= 0.477). The high score of the scale was the protective factor for the occurrence of anxiety (OR = 0.83, 95% confidence interval: 0.78~0.89, *p* < 0.001). See Table 2 for the detailed results.

**Table 2.** **Factors affecting anxiety in BC patients**.

Influencing factors	No. of studies	Test model	Heterogeneity test		Meta-analysis	
			*p*-value	I^2^ (%)	OR (95% CI)	*p*-value
Higher age	7	R	<0.001	78.7	0.90 (0.82, 0.98)	0.011
Lower education	3	R	<0.001	89.4	1.66 (0.58, 4.78)	0.347
Earlier cancer stage	4	R	<0.001	82.5	0.84 (0.42, 1.68)	0.621
Lower income	3	R	0.247	28.4	1.97 (1.39, 2.78)	<0.001
Rural residence	2	R	0.066	70.4	2.07 (0.84, 5.08)	0.112
LOT-R	2	R	0.477	0	0.83 (0.78, 0.89)	<0.001

R, Random-effects model; OR, Odds ratio; CI, Confidence interval.

#### Factors Influencing Depression

Age factor: Four studies analyzed the influence of age on the occurrence of depression [36, 37, 42, 45], with moderate heterogeneity between studies (I^2^ = 68.4%, *p *= 0.023). Older age was identified as a protective factor for depression (OR = 0.95, 95% confidence interval: 0.91~1.00, *p *= 0.037).

Education level factors: Three studies analyzed the impact of educational level on depression [15, 30, 33], with no significant heterogeneity between studies (I^2^ = 0%, *p *= 0.829). A low educational level was a significant risk factor for depression (OR = 2.59, 95% confidence interval: 1.80~3.73, *p* < 0.001).

Tumor staging factors: Three studies explored the role of tumor stage on depression [18, 22, 38], with no significant heterogeneity between studies (I^2^ = 0%, *p *= 0.759). Early tumor staging was a protective factor for depression (OR = 0.36, 95% confidence interval: 0.20~0.66, *p *= 0.001).

Income level factors: Seven studies analyzed the impact of income level on depression [23, 26, 29, 43, 44, 45, 46], with high heterogeneity between studies (I^2^ = 79.5%, *p* < 0.001). Higher income was a significant protective factor for depression (OR = 0.44, 95% confidence interval: 0.30~0.65, *p* < 0.001).

Marital status factors: Seven studies explored the impact of marital status on depression [18, 23, 28, 29, 37, 38, 44], with moderate heterogeneity between studies (I^2^ = 47.7%, *p *= 0.075). Marital breakdown (such as single, unmarried, divorced, separated) or married but living alone was a significant risk factor for depression (OR = 2.45, 95% confidence interval: 1.70~3.51, *p* < 0.001).

Pain factors: Two studies analyzed the impact of pain on depression [22, 25], with high heterogeneity between studies (I^2^ = 76.5%, *p *= 0.039). The presence of pain symptoms may increase the risk of depression, but the difference was not statistically significant (OR = 2.36, 95% confidence interval: 0.51~10.95, *p *= 0.273).

Family support factors: Two studies explored the effect of family support on depression [22, 25], with high heterogeneity between studies (I^2^ = 82.4%, *p *= 0.017). The effect of family support on depression was not statistically significant (OR = 0.10, 95% confidence interval: 0.00~15.05, *p *= 0.335).

Residence factors: Three studies analyzed the impact of residence on depression [26, 34, 43], with high heterogeneity between studies (I^2^ = 74.3%, *p* = 0.021). Living in rural areas was a significant risk factor for depression (OR = 2.01, 95% confidence interval: 1.06~3.80, *p* <0.032).

Life Orientation Test-Revised (LOT-R) Score: Two studies explored the impact of the scale score on depression [31, 32], with no obvious heterogeneity between studies (I^2^ = 0%, *p *= 0.887). A high scale score was a significant protective factor against depression (OR = 0.84, 95% confidence interval: 0.77~0.91, *p* < 0.001).

Helpless/hopeless coping factors: Two studies analyzed the impact of helpless/hopeless coping on depression [20, 31], with moderate heterogeneity between studies (I^2^ = 50.3%, *p *= 0.156). The use of helpless/unhopeful coping may increase the risk of depression, but the difference was not statistically significant (OR = 1.68, 95% confidence interval: 0.78~3.65, *p *= 0.188).

Concomitant factors: Two studies explored the effect of complications on depression [29, 32], with no obvious heterogeneity between studies (I^2^ = 0%, *p *= 0.332). The presence of complications was a significant risk factor for depression (OR = 1.81, 95% confidence interval: 1.31~2.49, *p* > 0.05).

Comorbidity: Two studies examined the effect of comorbidity on depression [29, 32], with no heterogeneity (I^2^ = 0%, *p *= 0.332). The presence of comorbidities was a significant risk factor for depression (OR = 1.81, 95% CI: 1.31–2.49, *p* < 0.001).

Social support factors: Three studies analyzed the impact of social support on depression [16, 18, 24], with no obvious heterogeneity between studies (I^2^ = 0%, *p *= 0.747). Lack of social support was a significant risk factor for depression (OR = 4.94, 95% confidence interval: 3.25~7.51, *p* < 0.001).

Factors of mental illness history: Three studies explored the impact of mental illness history on depression [20, 23, 37], with high heterogeneity between studies (I^2^ = 85.2%, *p *= 0.001). A history of mental illness was a significant risk factor for depression (OR = 6.00, 95% confidence interval: 1.61~22.43, *p *= 0.008). The detailed results are presented in Table 3.

**Table 3.** **Factors affecting depression in BC patients**.

Influencing factors	No. of studies	Test model	Heterogeneity test		Meta-analysis	
			*p*-value	I^2^ (%)	OR (95% CI)	*p*-value
Higher age	4	R	0.023	68.4	0.95 (0.91, 1.00)	0.037
Lower education	3	R	0.829	0	2.59 (1.80, 3.73)	<0.001
Earlier cancer stage	3	R	0.759	0	0.36 (0.20, 0.66)	0.001
Higher income	7	R	<0.001	79.5	0.44 (0.30, 0.65)	<0.001
Marital status	7	R	0.075	47.7	2.45 (1.70, 3.51)	<0.001
Pain	2	R	0.039	76.5	2.36 (0.51, 10.95)	0.273
Family Support	2	R	0.017	82.4	0.10 (0.00, 15.05)	0.335
Rural residence	3	R	0.021	74.3	2.01 (1.06, 3.80)	0.032
Higher LOT-R score	2	R	0.887	0	0.84 (0.77, 0.91)	<0.001
Helpless or despair coping style	2	R	0.156	50.3	1.68 (0.78, 3.65)	0.188
Comorbidity	2	R	0.332	0	1.81 (1.31, 2.49)	<0.001
Social Support	3	R	0.747	0	4.94 (3.25, 7.51)	<0.001
History of mental illness	3	R	0.001	85.2	6.00 (1.61, 22.43)	0.008

R, Random-effects model; OR, Odds ratio; CI, Confidence interval.

#### Sensitivity Analysis and Publication Bias

**Fig. 5.**
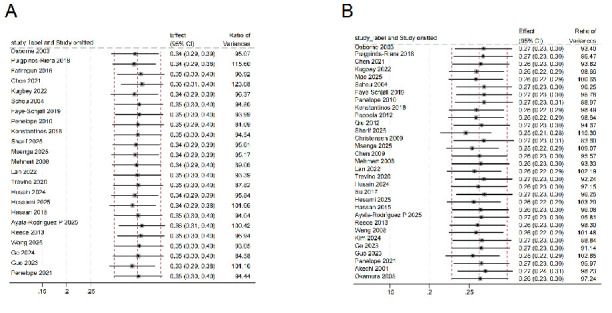
**Sensitivity analysis of the prevalence of anxiety and depression**. (A) Anxiety; (B) Depression.

The results of sensitivity analysis showed that after sequentially excluding any single study, the estimated point of anxiety and depression remained within the initial 95% confidence interval, indicating that the overall results of this study were robust (Fig. 5).

**Fig. 6.**
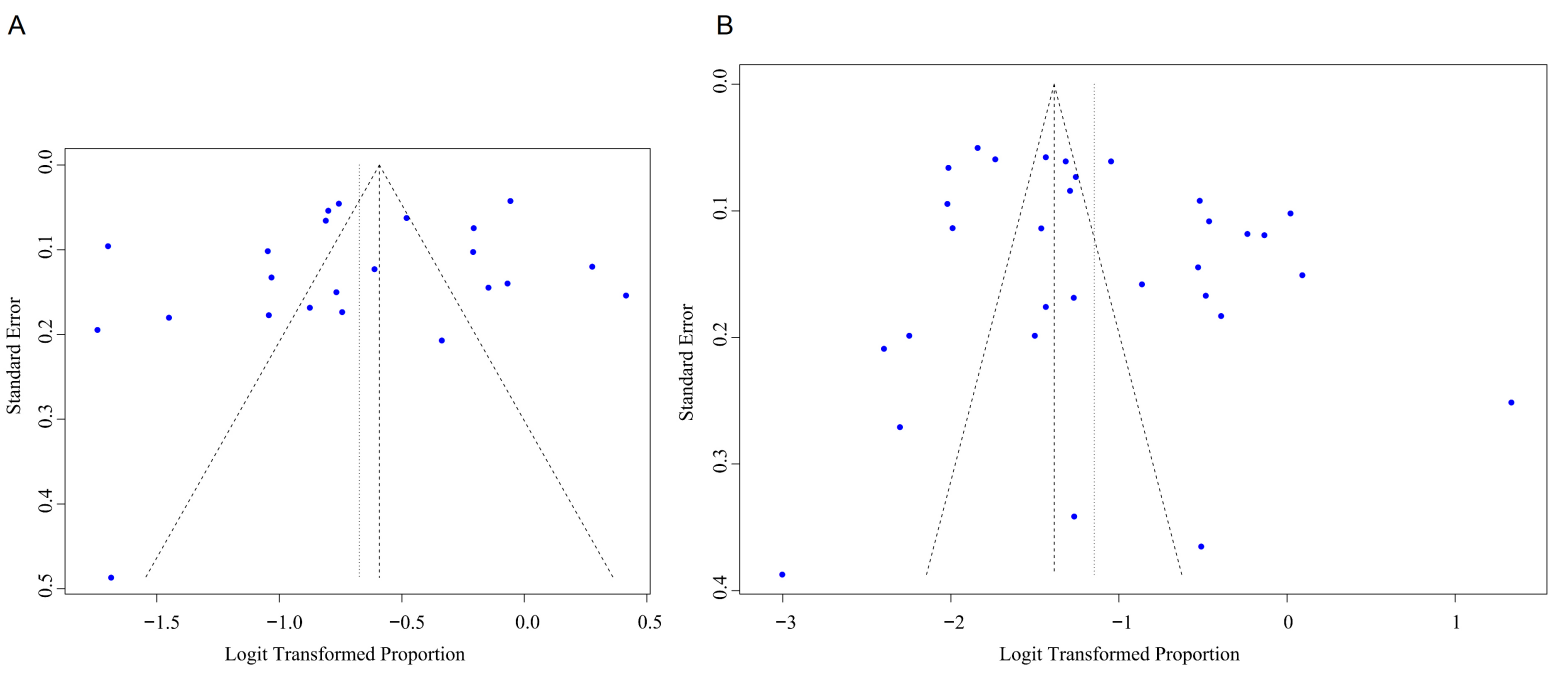
**Funnel plot for the prevalence of anxiety and depression**. (A) Anxiety; (B) Depression.

Visual inspection of the funnel plot combined with the results of the Egger’s test showed that obvious publication bias was detected in anxiety-related data and depression-related data (anxiety: t = 2.78, *p *= 0.01; depression: t = 2.21, *p *= 0.03) (Fig. 6).

**Fig. 7.**
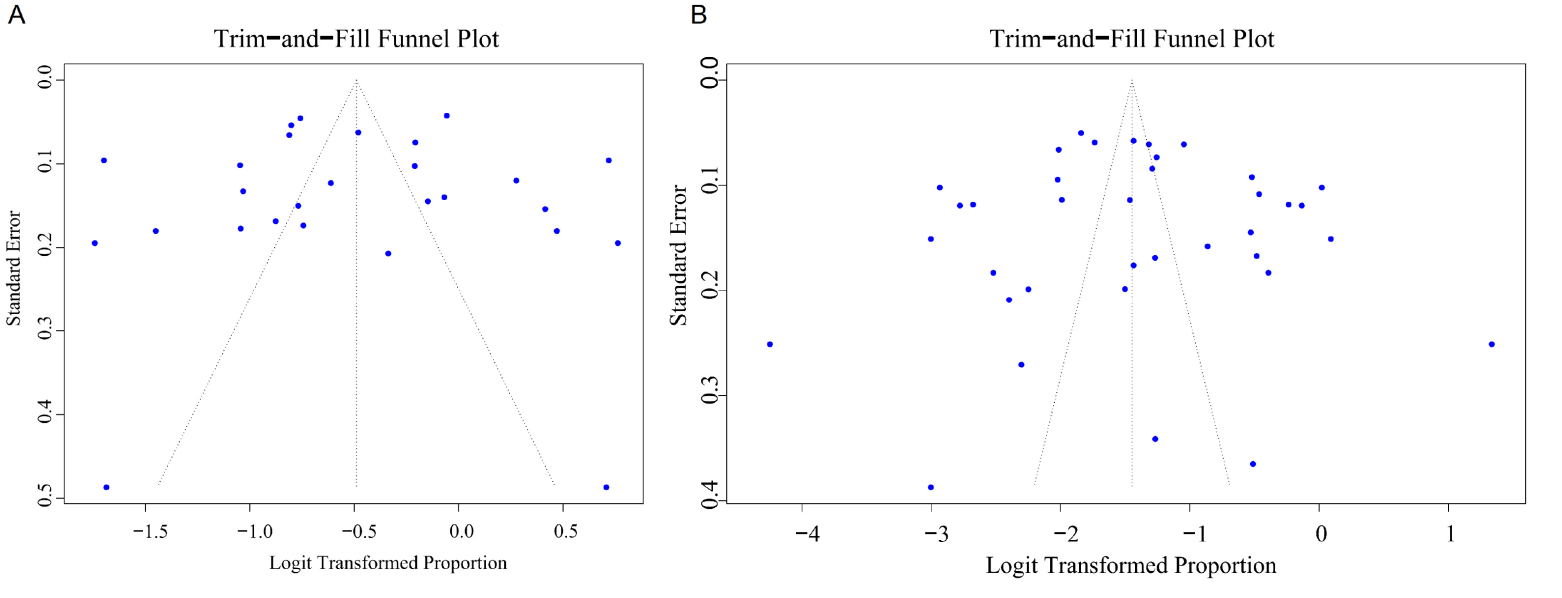
**The interpolated funnel plot**. (A) Anxiety; (B) Depression.

After correction by the trim-and-fill method, the estimated pooled prevalence increased slightly, but the overall trend remained unchanged (post-correction anxiety prevalence: 34.2%; pooled prevalence after depression correction: 26.4%) (Fig. 7), suggesting that the detected bias had a limited impact on the main conclusions of this study.

## Discussion

This meta-analysis included a total of 32 studies, involving 21507 BC patients. The results showed that the prevalence of anxiety symptoms in BC patients was 35% (95% CI: 30%–39%), the combined prevalence of depressive symptoms was 26% (95% CI: 23%–30%). The prevalence was consistent with the overall trend of cancer survivors [47], but significantly higher than the reported data of the general population [48]. In addition, subgroup analysis results indicated that factors such as low income, low education level, rural residence, lack of social support, history of mental illness and abnormal marital status (broken marital relationship/single/unmarried/divorced/separated/married living alone) significantly increase the risk of psychological problems in patients. Conversely, old age, high income level and early tumor staging were identified as relevant protective factors. The subgroup analysis found that there were significant differences in the prevalence of anxiety and depression corresponding to different assessment tools and different geographical regions. The study using DASS-21 reported the highest prevalence of depression, while the study using the structured clinical interview tool of the MINI was relatively low. Concise international neuropsychiatric interviews and interview tools based on the ICD diagnostic criteria are semi-structured or structured evaluation tools. Their diagnosis is based on clear, and they can more accurately identify anxiety and depression disorders that meet clinical diagnostic standards [49, 50]. However, in actual clinical work, limited by factors such as time, human resources and cost-effectiveness, DASS-21 and the HADS and other easy-to-operate and cost-effective screening tools are often used in clinical practice [51]. There are certain false positive and false negative rates in this kind of screening scale, which may lead to overestimation or underestimation of suspected cases. The results of geographical analysis showed that in studies conducted in Asia and Africa, the prevalence of depression was higher than that in Europe and North America. Differences in medical resources, cultural backgrounds, depression awareness and levels of economic development may affect the occurrence and diagnosis of anxiety and depression, which also highlights the uneven global distribution of mental health resources.

The meta-analysis of influencing factors showed that old age was a consistent protective factor for both anxiety and depression. This conclusion may be related to the psychological, social and physiological characteristics and disease-specific characteristics of elderly patients. Psychosocial and familial issues such as fertility concerns, childcare responsibilities, family demands, and financial pressures are more prominent in young adulthood than in later life [52]. Naik *et al*. [53] found that young adults aged 18–39 diagnosed with breast cancer were more likely to report psychosocial needs related to work/school, intimacy/sexuality, and finances, reflecting developmental challenges such as career progression, relationship maintenance, and economic stability [52]. These factors may contribute to more severe anxiety and depressive symptoms in younger patients. Regarding treatment, tumors in younger patients are often more aggressive, necessitating more intensive therapies with more severe side effects and longer recovery durations [52, 54]. Avis *et al*. [54] reported that illness intrusiveness is particularly pronounced in younger patients, with younger women reporting higher levels of disruption across all 16 assessed life domains (e.g., health, work, social relations, sexuality). Among patients under 45 years of age, “sexuality” was the most severely affected domain, which is closely associated with chemotherapy‑induced sexual dysfunction [54]. Such broad‑based disruption in life functioning may exacerbate emotional distress. Furthermore, social support and loneliness may mediate the relationship between age and emotional distress. Younger breast cancer survivors report higher levels of loneliness than older survivors, and loneliness is positively correlated with distress and fatigue [52]. Symptoms such as fatigue, loneliness, daytime sleepiness, and perceived stress are more severe and interrelated in younger patients, collectively worsening mental health outcomes, whereas older patients may experience these symptoms more independently or with lower intensity [54]. These findings underscore the need for age‑tailored psychosocial assessment and intervention, with particular attention to the needs of younger patients regarding fertility, career development, interpersonal relationships, and financial stability, to alleviate emotional distress and improve long‑term quality of life.

The meta-analysis identified income as a shared influencing factor for both anxiety and depression. Patients experiencing greater financial strain often face more difficulties in accessing high-quality medical services, psychosocial support and rehabilitation resources [55]. This substantial economic burden can contribute to heightened feelings of despair. Lower educational levels was associated with an increased risk of depression. Patients with lower education levels may have poorer coping mechanisms, health behaviours and access to medical resources [56]. Living in rural areas was also a risk factor for depression. Compared with urban areas, rural areas often face issues such as imbalanced distribution of healthcare resources, transport inconveniences, and insufficient dissemination of health knowledge [57]. Meanwhile, cancer patients and survivors in rural areas frequently experience a lack of mental health resources due to challenges like geographic isolation and lower income levels [58].

This meta-analysis showed that earlier tumor staging was a protective factor for the occurrence of depression. Patients with advanced tumors must face more complex treatment plans, more painful symptoms and more uncertain prognosis, which will bring a heavier psychological burden to patients [59, 60]. However, the correlation between tumor staging and anxiety did not reach statistically significant level, which may be because the triggers of anxiety are more immediate threats, such as upcoming surgery or chemotherapy, rather than simply by the severity of the disease itself [7].

Lack of social support was identified as a key risk factor for depression in BC patients (OR = 4.94). In this population, social support can play a dual role: on the one hand, it can cushion the impact of stress events; on the other hand, it can directly reduce depressive symptoms by cultivating positive coping strategies and improving the individual’s sense of control [61]. When BC patients lack sufficient social support, stress events will directly and comprehensively impact their psychological resilience, thus increasing the risk of depression. In this regard, abnormal marital conditions (broken marital relationship/single/unmarried/divorced/separated/married living alone) were also significantly associated with an increased risk of depression, which showed that the emotional support provided by the partner is a crucial psychological pillar during their cancer journey.

A history of mental illness emerged as the strongest risk factor for depression. Pre-existing mental health conditions may recur or worsen under the substantial psychological stress associated with a cancer diagnosis. The life Orientation Test-Revised (LOT-R) is a psychological measurement tool used to evaluate dispositional optimism. The higher scores on this scale indicate more positive expectations for the future and a tendency towards an optimistic cognitive style [62]. The results of this meta-analysis demonstrated that a high LOT-R scale score was significantly associated with a reduced risk of both anxiety and depression. This suggests fostering a positive cognitive mentality and a sense of hope may help reduce the prevalence of depression in BC patients.

Although the sensitivity analysis confirmed the robustness of the pooled results, and publication bias correction was observed, there was still a very high heterogeneity between the inclusion of the studies. This heterogeneity may stem from methodology variations, including differences in the assessment tools and cut-off values used to define anxiety and depression, which led to inconsistent case definitions and detection rates. The cross-sectional design adopted by most research institutes limits the validity of causal inference, and the sample representativeness in some investigations may also be restricted. Although this study has explored the source of heterogeneity through subgroup analysis and meta-regression, the original study did not fully report important covariates, such as specific treatment plan, the duration of the disease, the detailed type of complications and other important covariables, which restricts the further in-depth analysis of potential moderating factors. The results of the Egger’s test indicated the presence of publication bias in the depression-related data. Although the trim-and-fill correction only slightly altered the effect estimate, it cannot be ruled out that it has a certain impact on the overall combined estimate. Furthermore, the included literature spans a long period from 2001 to 2025, during which the treatment pattern of BC and psychosocial support measures may have evolved. This temporal change may have introduced time-related confounding factors.

## Conclusion

The pooled results of this meta-analysis suggest a potentially high prevalence of anxiety and depression in BC patients based on existing evidence. Several factors, including older age, higher income, and positive lifestyle tendencies, may serve as protective factors, while lower education level, lack of social support and a history of mental illness are associated with increased risk. Given the noted heterogeneity among studies, clinical recommendations should be interpreted with caution. Early psychological screening in identified high-risk groups could be considered, and prudent, evidence-based interventions targeting modifiable factors may help improve patient mental health.

## Availability of Data and Materials

All experimental data included in this study can be obtained by contacting the corresponding author if needed.
